# Technostress and Digital Competence Among Health Professionals in Swiss Psychiatric Hospitals: Cross-sectional Study

**DOI:** 10.2196/31408

**Published:** 2021-11-04

**Authors:** Christoph Golz, Karin Anne Peter, Thomas Jörg Müller, Jochen Mutschler, Sandra M G Zwakhalen, Sabine Hahn

**Affiliations:** 1 Department of Health Professions Bern University of Applied Sciences Bern Switzerland; 2 Private Clinic Meiringen Bern Switzerland; 3 Translational Research Center University Hospital of Psychiatry and Psychotherapy University of Bern Bern Switzerland; 4 Department of Health Services Research Care and Public Health Research Institute Maastricht University Maastricht Netherlands

**Keywords:** technostress, digital competence, psychiatry, health professionals, multiple regression

## Abstract

**Background:**

Psychiatric hospitals are becoming increasingly digitized because of the disruptive rise in technical possibilities. This digitization leads to new tasks and demands for health professionals, which can have an impact on technostress. It is unclear whether digital competence reduces technostress and how technostress affects health professionals’ mental and physical health.

**Objective:**

This study aims to assess the association between digital competence and technostress, considering individual characteristics and the association between technostress and long-term consequences for health professionals.

**Methods:**

Cross-sectional data from 3 Swiss psychiatric hospitals were analyzed using multiple linear regression. The dependent variables for the models were digital competence, technostress, and long-term consequences (intention to leave the organization or the profession, burnout symptoms, job satisfaction, general health status, quality of sleep, headaches, and work ability). One model was calculated for each long-term consequence. The mean scores for technostress and digital competence could range between 0 (*fully disagree*) and 4 (*fully agree*), where a high value for technostress indicated high technostress and a high value for digital competence indicated high digital competence.

**Results:**

The sample comprised 493 health professionals in psychiatric hospitals. They rated their technostress as moderate (mean 1.30, SD 0.55) and their digital competence as high (mean 2.89, SD 0.73). Digital competence was found to be significantly associated with technostress (β=−.20; *P*<.001). Among the individual characteristics, age (β=.004; *P*=.03) and profession were significantly associated with both digital competence and technostress. Technostress is a relevant predictor of burnout symptoms (β=10.32; *P*<.001), job satisfaction (β=−6.08; *P*<.001), intention to leave the profession (β=4.53; *P*=.002), organization (β=7.68; *P*<.001), general health status (β=−4.47; *P*<.001), quality of sleep (β=−5.87; *P*<.001), headaches (β=6.58; *P*<.001), and work ability (β=−1.40; *P*<.001).

**Conclusions:**

Physicians and nurses who have more interaction with digital technologies rate their technostress higher and their digital competence lower than those in other professions. Health professionals with low interaction with digital technologies appear to overestimate their digital competence. With increasing digitization in psychiatric hospitals, an increase in the relevance of this topic is expected. Educational organizations and psychiatric hospitals should proactively promote the digital competence of health professionals to manage expected disruptive changes.

## Introduction

### Background

Psychiatric hospitals are increasingly becoming digitized because of the disruptive rise in technical possibilities [1,2] and legal requirements, such as the obligation to use nationally shared electronic health records [3]. Moreover, the COVID-19 pandemic has underlined the need for additional digital services such as telemedicine or remote monitoring in mental health to avoid social exclusion through lockdowns or because of living situations in remote regions [4,5]. Health professionals are thus increasingly confronted with digital technologies for clinical practice, interaction with patients, and administrative tasks.

Therefore, digitalization creates new tasks for health professionals and places demands on them that are not part of their education and training. These include, for example, the management of data privacy [1] or digital competences to enhance appropriate patient communication via internet [6]. In addition, new tasks make demands such as increasing time spent with documentation [7,8] or with low usability electronic health records [9] and technical support among colleagues [10], which were previously beyond the scope of work of health professionals.

The demands for digital competences and associated changes in the role of health professionals also require a change in the perception of and attitude toward digital resources in everyday work [11]. Consequently, this transformation may have a stress-inducing effect on health professionals, especially because psychiatric health professionals tend to be hesitant regarding new technologies because of the expected deleterious effects on the relationship between health professionals and patients [12,13]. For example, they may feel more disturbed by the digitization of their daily work than their colleagues in settings that are traditionally more digitized, such as acute care with intensive care units.

The phenomenon called technostress is “a reflection of one’s discomposure, fear, tenseness and anxiety when one is learning and using computer technology” [14]. The term was introduced in 1984 by Brod [15] as “a modern disease of adaptation caused by an inability to cope with the new computer technologies in a healthy manner” during the rapid emergence of technology in everyday life. Studies on technostress among health professionals are scarce [16,17]. A recent study revealed that psychiatric health professionals experience a moderate level of technostress [16].

Technostress is known to have an effect not only on the working life of professionals [10], such as reduced job satisfaction [18,19], but also on their private life, such as psychophysiological reactions such as headaches and fatigue [20,21] or burnout symptoms [22]. Exposure to stress-inducing technology can even result in reduced ability to work and an intention to leave the job, which could exacerbate the already-existing shortage of health professionals [23].

An important factor in technostress is expected to be an individual’s digital competence, as higher digital competence has been identified as having a mitigating association with technostress [10,24]. However, it was found that professionals with high digital competence tended to feel particularly stressed by the nonavailability or unreliability of the technologies used at work [24]. Research on digital competence among health professionals has quite a strong focus on the knowledge and skills of using digital technologies at work [25] or specific subgroups in nursing, such as nurse leaders [26,27]. The TIGER Nursing Informatics Competencies Model, for example, consists of 3 parts: basic computer competences (eg, using the computer and managing files), information literacy (eg, evaluating information and its sources critically), and information management (eg, using electronic health records) [25]. However, additional factors, such as attitude, motivation, and experience of using digital technologies, are also thought to be relevant in the context of digital competence. A recent review of research on health professionals’ digital competence summarized the key areas of this competence as “sufficient knowledge and skills [...], social and communication skills [...], motivation and willingness [...] and support for positive experiences in digitalization” [28]. Therefore, besides insufficient knowledge and skills for proper implementation and use of digital technologies, a lack of motivation and prejudice against digitalization are, for example, associated with reduced technology use. Moreover, health professionals must adapt their communication style, depending on whether they communicate face to face or via telemedicine [28]. Therefore, behavioral determinants are crucial for enhancing digital competence in addition to knowledge and skills [29].

Unfortunately, findings on digital competence and its association with technostress are not specific to health professionals in psychiatric hospitals. However, it is especially important for health professionals that information on their digital competence and technostress is needed, as they are considered to be reluctant adapters of digitization, despite increasing calls for adaptation to new tasks and requirements to keep up with their profession. These contradictions of reluctance and ongoing change need to be addressed at an early stage.

### Objective

This paper, therefore, aims to answer the following research questions:

1. How do health professionals in psychiatric hospitals rate their digital competence?
2. How do health professionals in psychiatric hospitals rate their technostress?
3. What is the association between health professionals’ digital competence and their technostress, considering the individual characteristics of health professionals?
4. What is the association between technostress and long-term consequences for health professionals?

## Methods

This cross-sectional study was conducted in 3 psychiatric hospitals in the German-speaking part of Switzerland as part of the Work-Related Stress Among Health Professionals in Switzerland (STRAIN) study [23]. This study is based on a cluster randomized controlled trial (Clinical Trials registration NCT03508596) consisting of 3 measurements (baseline, first, and second) and investigating work-related stress among health professionals in Switzerland.

### Sample and Recruitment

The study sample of the STRAIN study included acute care and rehabilitation hospitals, psychiatric hospitals, nursing homes, and home care organizations. Detailed information on the STRAIN study sample has been published elsewhere [23]. For this study, a request to participate was sent to 12 psychiatric hospitals that had already participated in the STRAIN study. The internal coordinators of the psychiatric hospitals were contacted by email and asked whether their institution’s health professionals might participate in this study, which would focus on technostress and digital competences. The project was then presented to decision makers at the psychiatric hospitals. Health professionals from the following work categories were included in this study: nursing staff, physicians, psychologists, medical therapeutic professionals, and social workers. Participants who labeled themselves as *researcher* or secretariat in the additional free text field were excluded. Overall, 1767 health professionals were eligible for participation in the study.

### Data Collection

The study was conducted along with the second measurement of the STRAIN study between June and September 2020. The questionnaires for health professionals from the institutions that had agreed to participate were expanded to include topic-specific scales measuring technostress and digital competence.

The internal coordinator of the participating psychiatric hospitals disseminated the information for the participants and the survey to health professionals. Participation in the study was possible via paper or web-based questionnaires in German. For the paper questionnaires, a prestamped envelope was enclosed to return the questionnaire to the project team. For the web-based questionnaire, the link to the web-based survey using SurveyMonkey and UmfrageOnline was either sent individually by email or published on the organization’s intranet by the coordinator. A reminder to complete the questionnaire was sent electronically or on paper 2 weeks afterward by the internal coordinator.

### The Questionnaires

The 3 questionnaires used in this study comprised a technostress questionnaire [24], an in-house-developed digital competence questionnaire, and the STRAIN questionnaire [23]. The questionnaires were estimated to take 45 minutes overall to complete.

### Technostress Questionnaire

For the measurement of technostress, the scale created by Gimpel et al [24] was used. The scale, which shows satisfactory reliability (Cronbach α=.91), is based on the technostress model of Ayyagari et al [30]—a model widely used in research on technostress. It consists of 12 items using a 5-point Likert scale, with the end points 0 (*fully disagree*) and 4 (*fully agree*). For interpretation of the data, the mean score was calculated (min=0; max=4), where a high score indicates high technostress. The questionnaire covers the following 12 items, which are derived from the theory’s dimensions: uncertainty (ongoing changes lead to uncertainty and constant learning), insecurity (feeling threatened about losing one’s job), unreliability (unreliability of technology used), overload (technology forces users to work faster and longer), invasion (employees can be reached anytime), complexity (users feel inadequate regarding their competences), performance control (feeling of being monitored and compared), ambiguity of the role (technical problems must be solved by oneself), interruptions (malfunctions and unstable systems), nonavailability (lack of technology that can reduce workload), no sense of achievement (feeling of lack of progress at work), and invasion of private life (feeling one’s private life is affected).

### Digital Competence Questionnaire

To measure digital competence among health professionals, no suitable and compact questionnaire was available that focused on the 5 key areas of digital competence (knowledge, skills, communication, experience, and attitude) for health professionals [28]. Moreover, to not lengthen the already-long questionnaire excessively, thereby negatively influencing the response rate, a short self-assessment scale measuring digital competence was needed. Therefore, for each of the 5 key areas, an item was developed in-house. The 5 items covered the following topics: knowledge (eg, one’s own knowledge of digital technologies at work), skills (confidence in using digital technologies at work), communication (eg, confidence in communication using digital technologies at work), motivation (eg, motivation to use digital technologies in everyday work), and attitude (eg, attitude toward potential improvements through digital technologies at work). Items were scored on a 5-point Likert scale ranging from 0 (*fully disagree*) and 4 (*fully agree*). For interpretation, the mean score was calculated (min=0; max=4), with a high score again indicating high digital competence.

The single items of digital competence were tested for construct validity by conducting exploratory factor analysis and reliability tests. The requirements for factor analysis were met with item correlations above 0.3 and a significant Bartlett test of sphericity (*χ^2^*_4_=39.4, *P*<.001) and the Kaiser-Meyer-Olkin (KMO) measure of sampling adequacy with acceptable values above 0.6 (KMO=0.81). A scree plot was used to test for loadings on one factor. The reliability test for the 5 developed items on digital competence revealed satisfactory internal consistency (Cronbach α=.87; Multimedia Appendix 1).

### STRAIN Questionnaire

**Figure 1:**
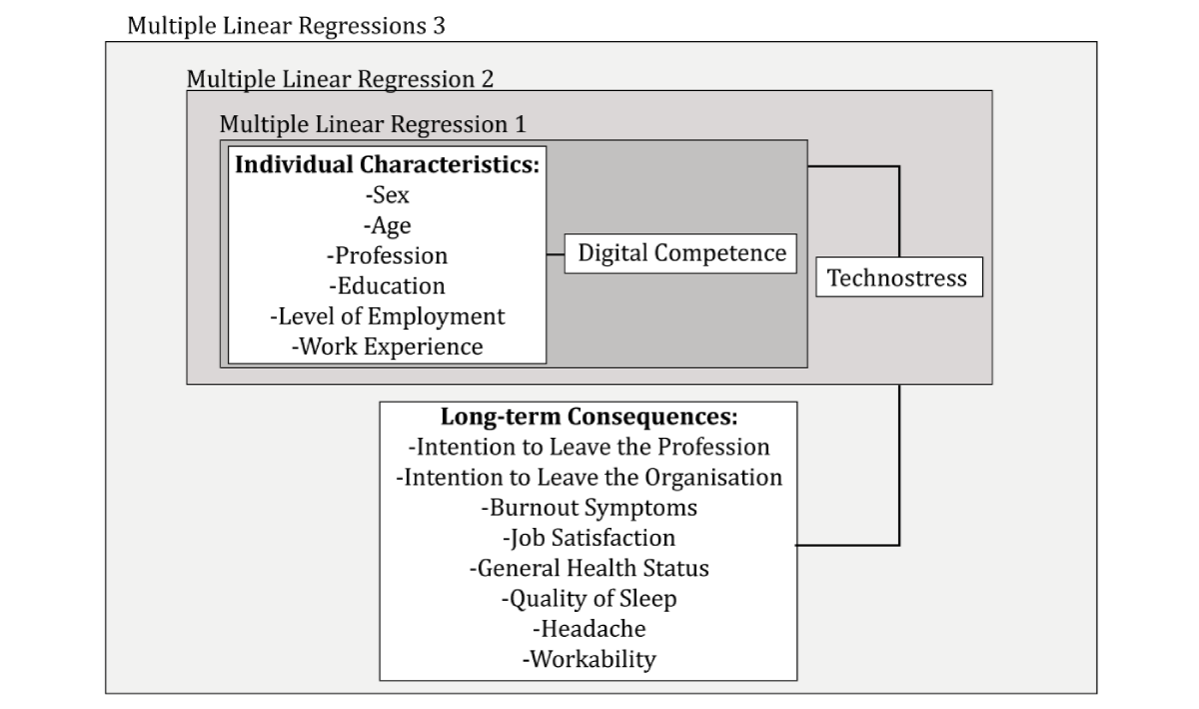
Scales used for the multiple linear regression models.

The outcome variables (Figure 1) for long-term consequences stem from the STRAIN questionnaire [23,31], which comprises well-known, valid, and reliable scales such as the Copenhagen Psychosocial Questionnaire (COPSOQ) [32], the self-rated general health status [33], the Nurses’ Early Exit study questionnaire [34], the von Korff questionnaire [35], and the workability index [36]. The scores from the COPSOQ, the Nurses’ Early Exit study questionnaire, the von Korff questionnaire, and the general health status ranged from a value of 0 (*do not agree at all*) to 100 (*fully agree*) or from 0 (*worst imaginable health state*) to 100 (*best imaginable health state*) for the general health status and from 0 (*no influence*) to 100 (*could no longer perform activity*) for the von Korff questionnaire. The COPSOQ scale scores were included if at least half of the items had no missing values [37]. The total score of the workability index questionnaire ranged from 7 (*minimum working capacity*) to 49 (*maximum working capacity*).

### Data Analysis

The analysis was conducted using R version 3.6.1 [38] and included descriptive statistics for technostress and digital competence. Multiple linear regression models were calculated using the MASS package [39]. The predictor and outcome variables were chosen to cover the dimensions of the DSM [24]. The model describes the correlation between technostress, inhibitors of technostress, and consequences of technostress. Furthermore, individual characteristics (eg, age, education, and sex) were added to the model, as they have been identified as relevant predictors elsewhere [10]. To answer the research questions, multiple linear regressions were conducted (1) with digital competence as the outcome and individual characteristics as predictors; (2) with technostress as the outcome and individual characteristics and digital competence as predictors; and (3) with long-term consequences as outcome variables and technostress, digital competence, and individual characteristics as predictors (Figure 1). For each of the following long-term consequences, a separate multiple linear regression was calculated: intention to leave the organization [23], intention to leave the profession [23], burnout symptoms [32], job satisfaction [32], general health status [33], quality of sleep [34], headache [35] and workability [36].

To minimize the effect of internal dropouts, missing data were filled in based on multiple imputation expecting data to be missing completely at random, using the MICE package [40]. To test for multicollinearity, the variance inflation factor was computed (1.06-1.70), which is regarded as acceptable to proceed if variables show values less than 3 [41]. The assumption of heteroskedasticity was tested using the Breusch-Pagan test. This was met for multiple linear regressions. Therefore, SEs, *P* values, and CIs were bootstrapped (*r*=999, bias corrected and accelerated, 95% CI). A stepwise model selection was conducted for the multiple linear regressions based on the Akaike information criterion [42].

### Ethical Considerations

The local Swiss ethical board confirmed that the study did not warrant a full ethical application and did not fall under the Swiss Federal Act on research involving human beings (Req-2020-00179). The participants were professionals and could take responsibility for their own participation. They received written information before the start of the study regarding the subject, aim, and voluntary nature of their participation. Filling in the questionnaire was counted as informed participation. The data were gathered anonymously and could not be traced back to individual participants.

## Results

In total, 493 health professionals participated in the study, corresponding to a response rate of 27.9% (493/1767). Among the participants, 60% (296/493) were nurses, 12.3% (61/493) were psychologists, 11.1% (55/493) were social workers, 8.7% (43/493) were physicians, and 7.7% (38/493) were medical-therapeutic professionals. The mean age of the participants was 41 (SD 12.33) years, and the majority were female (349/493, 71%). For technostress, health professionals reported a moderate mean score of 1.30 (SD 0.55). Nursing staff (mean 1.41, SD 0.54) and physicians (mean 1.41, SD 0.54) had the highest scores among the professions included, followed by medical-therapeutic professionals (mean 1.23, SD 0.60), social workers (mean 1.15, SD 0.57), and psychologists (mean 0.95, SD 0.40). Health professionals rated their digital competence high, with a mean score of 2.82 (SD 0.76): social workers were found to have the highest score (mean 3.18, SD 0.57), followed by medical-therapeutic professionals (mean 2.90, SD 0.84), psychologists (mean 2.89, SD 0.73), physicians (mean 2.82, SD 0.66), and nurses (mean 2.71, SD 0.78).

### Technostress

Table 1 summarizes the results of the multiple linear regression, with technostress as the outcome variable. The regression model was shown to be significant *F*_5,487_=19.81 (*P*<.001) and explained 20% of the variance (*R*^2^). Being a physician (β=.22; *P*=.03) or a nurse (β=.17; *P*=.02) was shown to have an increasing association with technostress, compared with being a social worker (intercept), whereas being a psychologist was negatively associated with technostress (β=−0.23; *P*=.01). Digital competence was also negatively associated with technostress (β=−0.20; *P*<.001). This means that an increase in digital competence of 1 point results in a decrease in technostress by −0.20 points of the mean score.

**Table 1:** Multiple linear regression with technostress as the outcome [observations N=493; technostress: 0 (no technostress) to 4 (high technostress)].

Coefficient	β	SE	*t* value (*df*)	*P* value	95% CI
Intercept	1.63	0.15	10.86 (487)	<.001	1.62 to 1.64
Age	.004	0.002	2.21 (1)	.03^a^	0.004 to 0.004
Physicians	.22	0.10	2.22 (1)	.03^a^	0.22 to 0.23
Psychologists	−.23	0.09	−2.53 (1)	.01^a^	−0.24 to −0.23
Nurses	.17	0.07	2.30 (1)	.02^a^	0.16 to 0.17
Digital competence	−.20	0.03	−6.71 (1)	<.001	−0.21 to −0.20

^a^With bootstrap.

### Digital Competence

The multiple linear regression with digital competence as the outcome was shown to be significant *F*_6,486_=10.47 (*P*<.001) and explained 13% of the variance (*R*^2^). Being male was shown to be positively but not significantly associated with digital competence (β=.11; *P*=.15). In addition, the level of employment was positively associated with digital competence (β=.006; *P*<.001). Age was negatively associated with digital competence (β=−0.014; *P*<.001), meaning that digital competence decreased marginally with increasing age (Table 2).

**Table 2:** Multiple linear regression with digital competence as outcome [observations N=493; digital competence: 0 (no digital competence) to 4 (high digital competence)].

Coefficient	β	SE	*t* value (*df*)	*P* value	95% CI
Intercept	3.25	0.21	15.52 (486)	<.001	3.24 to 3.26
Sex (male)	.11	0.08	1.45 (1)	.15^a^	0.10 to 0.11
Age	−.014	0.003	−5.29 (1)	<.001	−0.01 to −0.01
Level of employment	.006	0.002	3.21 (1)	<.001	0.006 to 0.006
Physicians	−.46	0.15	−3.11 (1)	<.001	−0.47 to −0.45
Psychologists	−.26	0.13	−1.92 (1)	.06^a^	−0.26 to −0.25
Nurse	−.48	0.11	−4.55 (1)	<.001	−0.49 to −0.48

^a^With bootstrap.

### Long-Term Consequences

The results of the multiple regression models with long-term consequences as the outcome variables are shown in Multimedia Appendices 2 and 3. The models indicate that the independent variables predict the outcome *burnout symptoms* as best (*R*^2^=0.16, *F*_10,482_=9.28; *P*<.001), followed by *intention to leave the organization* (*R*^2^=0.15, *F*_13,485_=6.37; *P*<.001) and *job satisfaction* (*R*^2^=0.15, *F*_12,480_=5.28; *P*<.001). *General health status* turned out to have the lowest explanatory power with the included predictor variables (*R*^2^=0.06, *F*_3,489_=9.88; *P*<.001).

In all models, technostress was significantly associated with outcome variables. The highest impact was found for *burnout symptoms*, with an increase of 10.32 (*P*<.001) associated with an increase in technostress of 1 point. Technostress was also positively associated with *headache* (β=6.58; *P*<.001) and the outcomes *intention to leave the profession* (β=4.53; *P*=.02) and *intention to leave the organization* (β=4.53; *P*<.001). Moreover, technostress was negatively associated with *job satisfaction* (β=−6.08; *P*<.001), *general health status* (β=−4.47; *P*<.001), *quality of sleep* (β=−5.87; *P*<.001), and *workability* (β= −1.40; *P*<.001).

The predictor variable, digital competence, was included in 6 of the 8 models. The effect of digital competence was lower than that of technostress. Digital competence was positively associated with *quality of sleep* (β=4.19; *P*<.001), *job satisfaction* (β=2.26; *P*=.02), and *workability* (β=.79; *P*=.002). When interpreting the results, attention must be paid to the possible scores of the outcome variables. Thus, an increase in digital competence of 1 point leads to an increase in workability of 0.79, whereby workability can range from 7 to 49. An increase of 1 point in digital competence leads to an increase of 2.26 points in job satisfaction on a possible range of 0 to 100.

## Discussion

### Principal Findings

Health professionals in psychiatry rate their technostress as moderate, and their digital competence as high. Higher digital competence was also significantly associated with lower technostress. Individual characteristics differ in their relevance to the models. The age of health professionals is significantly associated with technostress and digital competence. Older health care professionals appear to experience higher technostress and perceive themselves as having lower digital competence. Physicians and nurses appear in the models to have higher technostress and lower competence compared with the other professions surveyed. Being a nurse was shown to have the highest estimates across all outcomes.

To answer the question of the association between technostress and long-term outcomes of health professionals, it should be noted that technostress has a nonnegligible impact on long-term consequences, such as burnout symptoms, job satisfaction, and headache. Thus, technostress has a measurable association with the mental and physical health of health professionals. In addition, technostress promotes the intention to leave the organization or the profession.

### Comparison With Prior Work

The significant association of digital competence with technostress is in line with another study in which *computer self-efficacy* (ie, digital competence) is described as an antecedent of technostress [10]. This association highlights the potential of enhanced digital competence to reduce technostress. However, the β values in the technostress model were equally high for the professions, which could mean that health professionals need to interact with digital technologies to varying degrees at work.

Interestingly, physicians and nurses who are known to have higher technostress [16] and thought to have more interaction with digital technologies than other health professionals were shown to have lower digital competence. This is in contrast with the findings of Kuek and Hakkennes [43], who found that health professionals with high-frequency digital technology use also showed higher digital competence. However, they argued that the organization in which the study took place was digitized more than organizations in comparable studies. One reason for the reported lower digital competence in this study could be past experience with digital technologies rather than a lack of knowledge and skills. Past experiences could have been negative because of a lack of *suitable rooms or technical equipment and failing support systems* [28]. Furthermore, it raises the question of whether health professionals who have experienced fewer negative interactions rate their digital competence higher because of the absence of digital technologies at work. These results are somewhat at odds with the results of other studies in which people who have little contact with digital technologies show higher levels of technostress because they lack opportunities to adapt and develop their own skills in using them [24]. This phenomenon could be explained by the Dunning-Kruger paradigm for this study. Studies “repeatedly show that people with little expertise [in the specific field] often grossly overestimate how much they know and how well they perform” [44]. However, this study does not provide any insights into the extent of interactions of health professionals with digital technologies.

Furthermore, lower digital competence (ie, computer proficiency) has been found to be a barrier to successful implementation of electronic health records in psychiatric hospitals [11]. This would imply that Swiss psychiatric hospitals have a good precondition for the successful implementation of digital technologies, as the digital competence of health professionals was rated high. However, being an active user of electronic health records was one of the inclusion criteria for the study, which means that participants self-rated their digital competence by having sufficient experience of interaction with digital technologies. According to Staggers et al [45], there are 4 different levels of digital competence for nurses. They propose that experienced nurses (level 2) are “highly skilled in using information management and computer technology skills” [45]. This expands the understanding of the core competences necessary for consideration as an experienced professional and places a requirement on educational organizations and psychiatric hospitals to support health professionals in fulfilling this aim. Recent findings also highlight the importance of leaders investing in technical support for their employees, such as “receiving low support in learning and using digital tools” [46], which is expected to contribute to enhanced digital competence [28].

Concerning gender, there was no strong evidence as to whether males or females were more affected by technostress. However, the model for digital competence indicated that being male was slightly but not significantly associated with digital competence (*P*=.15). One reason for this result could be that the clear majority of participants were female (71%), which could have led to an underestimation of the potential difference between the sexes. Regarding the technical support described earlier, females seem to compensate for their lower digital competence by relying on the organization’s helpdesk, whereas males tend to exchange expertise [47]. This implies that health organizations might want to invest in a low-threshold helpdesk and train health professionals with an affinity for digital technologies to become peer supporters.

Evidence for the effects of individual characteristics is inconsistent, particularly with respect to age and sex [10]. This study contributes to the discussion by indicating that age is a relevant predictor of both technostress and digital competence. In terms of digital competence, the results of this study appear to confirm that younger health care professionals perceive themselves as having higher digital competency [48]. However, recent findings, albeit nonspecific to the health care setting, indicate that females tend to be more affected by technostress [49]. In this respect, a possible effect of sex should be considered in future studies that focus on health care professionals. If it turns out that women are more affected by technostress in the health care system, the intended measures must take this possible precondition into consideration.

In terms of the association between technostress and its long-term consequences, other findings from other sectors underline that higher technostress leads to higher intention to leave the profession or organization and lower job satisfaction [50]. Furthermore, additional influencing factors in health care appear to have a more important impact on long-term consequences for health professionals, such as work-private life conflict or quantitative demands at work [23,51]. However, some aspects of private life conflicts are incorporated into the technostress scale used. One of the themes of technostress is *techno-invasion*, which measures the self-perceived aspect that one can be reached at any time. Also, the theme *invasion of private life* is part of the technostress scale, assessing the feeling that one’s private life is affected by digital technologies at work. Although these aspects are included in the technostress scale, the findings in this study do not reach the explained variance of the study indicated above. Therefore, it seems that digital technologies do not currently play a vital role in the context of private life conflicts among health professionals in psychiatric hospitals.

In view of the fact that the Swiss health care system is still only partly digitized in terms of international comparison [52] and that psychiatry is not expected to lead the way in digitization, these findings seem logical. However, with a future increase of digitization in psychiatric hospitals [53], the topic’s relevance is expected to rise. For example, a recent study described the empowerment and enslavement paradox of digital technologies for surgeons [54]. The study highlights the issue that with an increase in possibilities because of digital technologies, the danger of misuse increases, which negatively impacts the outcomes of health professionals and patients. The implication for psychiatric hospitals is, therefore, that technostress is not a major issue at the moment. However, psychiatric hospitals are encouraged to invest in monitoring the digital competence of their health professionals, especially along with the implementation of digital technologies, and offer suitable training to their employees. Furthermore, decision makers should involve health professionals in the development and implementation of digital technologies, as involvement has been identified as crucial for positive experiences with digital technologies, increasing motivation toward innovations and dismantling prejudices [10]. Health professionals must recognize that they are going to face digitization at their workplace. However, because many health professionals have a rather reserved attitude toward digital technologies at work, decision makers should approach this process thoughtfully.

### Strengths and Limitations

This study contributes to the emerging topic of technostress among health professionals in a psychiatric setting. It provides first insights into the association of digital competence with technostress and the association of the two with long-term consequences. This study enriches the discussion on the potential influence of individual characteristics, such as age, sex, profession, and education. Furthermore, a digital competence scale with satisfactory properties was developed and evaluated in this study. This scale is made available to the community for use in further research (Multimedia Appendix 1).

However, this study had several limitations. First, convenience sampling was performed. Of the 12 psychiatric hospitals invited, only 3 agreed to participate. It cannot be excluded that psychiatric hospitals whose staff generally experience lower technostress agreed to participate because they were more sensitized to the topic. In addition, the sample did not reflect the typical distribution of health professionals in Swiss psychiatric hospitals. In this study, physicians were underrepresented (9%), compared with the usual proportion of 17% [55]. This might be because physicians are increasingly reluctant to participate in surveys for reasons such as information overload, survey fatigue, or privacy concerns [56]. In addition, a response rate of 27.9% (493/1767) is considered low but rather common for web-based surveys with health professionals [57,58]. Unfortunately, forecasts indicate even lower average response rates soon [59]. Furthermore, participants could decide to use either a paper or web-based questionnaire. The comparability of paper and web-based questionnaires has been discussed in the literature. Psychological factors, such as mood state or fatigue during the inquiry, can have an impact on responses and can be influenced by *environmental stimuli or distractions* [60]. Especially in health care organizations in which the number of computers on the wards is limited and no quiet place is available to withdraw, this could have had a deleterious effect on responses. In addition, one organization opted exclusively for web-based inquiry. Staff members who feel highly stressed by digital technologies could have been excluded by this decision because they did not want to use the computer unnecessarily for longer than was required by their work. Moreover, no causal conclusions can be drawn, as this study used cross-sectional data. These implications must be considered when interpreting the results.

### Conclusions

Health professionals in Swiss psychiatric hospitals experience moderate technostress at work. They rated their digital competence as high. It might be that health professionals with little interaction with digital technologies at work overestimate their digital competence. Therefore, to generate reliable results on this hypothesis in the future, the degree of digitization of the organization and the degree of contact with digital technologies at the individual level must be additionally assessed. In this context, research should evaluate whether self-rated digital competence corresponds to an objective assessment of digital competence at work, which would contribute to further development of the measurement tool for digital competence.

Technostress has been shown to have a relevant association with long-term consequences for staff, especially those with burnout symptoms. Further digitization in psychiatric hospitals is expected to have an increasing impact on the technostress experienced. Additional digital competence will be needed as an inhibitor of technostress for health professionals to sustainably cope with technostress and, thus, lower the risk of long-term consequences.

Health professionals and professionals in educational organizations do not yet recognize the need for future digital competences. Health and educational organizations are responsible for the adequate preparation of future health professionals; however, this should include training aimed at digital competence.

Psychiatric hospitals can draw conclusions based on these results. As digital competence significantly reduced technostress, further in-house education to promote digital competence should be established. Furthermore, the duties of younger health professionals could be extended to support older health professionals in managing digital technologies at work. Mutual support is demonstrably conducive to acquiring new competences and strengthening the sense of community in the team. However, this presupposes that such a duty is appropriately appreciated and remunerated.

Psychiatric hospitals in Switzerland are still in their early days in terms of the impact of digital technologies on health professionals. The necessary digital competences will emerge as the digitization process progresses. Researchers must continue to monitor this development and generate recommendations for measures to reduce technostress and develop suitable educational content from intervention studies.

## Appendix

Multimedia Appendix 1 Questionnaire Digital Competence.

Multimedia Appendix 2 Multiple linear regression models with long-term consequences as outcomes part 1 (observations N=493).

Multimedia Appendix 3 Multiple linear regression models with long-term consequences as outcomes part 2 (observations N=493).

## Abbreviations

COPSOQ: Copenhagen Psychosocial Questionnaire
KMO: Kaiser-Meyer-Olkin
STRAIN: Work-Related Stress Among Health Professionals in Switzerland
