# Supplementary material for: Technostress and Digital Competence Among Health Professionals in Swiss Psychiatric Hospitals: Cross-sectional Study
Source: JMIR Ment Health. 2021 Nov 4;8(11):e31408. doi: 10.2196/31408 (PMC8603177; doi:10.2196/31408)
Supplement: Multimedia Appendix 1 [file mental_v8i11e31408_app1.docx]

Questionnaire Digital Competence

1. In general, I rate my knowledge of digital technology as satisfactory.
2. I feel confident about finding relevant information using digital technology.
3. I feel confident about sharing information using digital technology.
4. I like to use digital technology at work.
5. I believe that digital technology has noticeable benefits for the quality of care.
