# Supplementary material for: Technostress and Digital Competence Among Health Professionals in Swiss Psychiatric Hospitals: Cross-sectional Study
Source: JMIR Ment Health. 2021 Nov 4;8(11):e31408. doi: 10.2196/31408 (PMC8603177; doi:10.2196/31408)
Supplement: Multimedia Appendix 2 [file mental_v8i11e31408_app2.docx]

Table 3: Multiple linear regression models with long-term consequences as outcomes part 1 (observations n=493)

|  | Intention to leave the profession^a^ | | Intention to leave the organization^a^ | | Burnout symptoms^a^ | | Job satisfaction^a^ | |
| --- | --- | --- | --- | --- | --- | --- | --- | --- |
|  |  | |  | |  | |  | |
|  | R2 = 0.08, F(7,485) = 6.37, *P* < .001 | | R2 = 0.15, F(13,485) = 6.37, *P* < .001 | | R2 = 0.16, F(10,482) = 9.28, *P* < .001 | | R2 = 0.15, F(12,480) = 5.28, *P* < .001 | |
|  | β | se | β | se | β | se | β | se |
| Intercept | 24.84*** | 7.22 | -1.01 | 12.72 | 16.34 | 10.87 | 76.77*** | 8.95 |
| Technostress | 4.53** | 1.96 | 7.68*** | 2.02 | 10.32*** | 1.65 | -6.08*** | 1.38 |
| Digital Competence | -2.61 | 1.37 |  |  | -2.46* | 1.18 | 2.26* | 0.02 |
| Sex: male |  |  | -4.96* | 2.32 | -3.55 | 1.90 |  |  |
| Age | -0.25** | 0.08 | -0.26 | 0.14 | -0.27*** | 0.07 | 0.21*** | 0.06 |
| Level of employment |  |  |  |  | 0.19*** | 0.48 |  |  |
| Work experience |  |  | -0.26 | 0.15 |  |  |  |  |
| Physicians | 2.23 | 4.35 | 2.65 | 6.56 |  |  | 4.11 | 4.36 |
| Psychologists | 1.98 | 3.97 | 2.79 | 5.67 |  |  | 0.67 | 3.77 |
| Nurses | 8.78** | 3.18 | 14.05*** | 3.57 |  |  | -5.79* | 2.32 |
| Medical therapeutic professionals | -1.99 | 4.49 | 1.05 | 4.85 |  |  | -3.48 | 3.19 |
| Education: secondary level |  |  | 9.16 | 11.67 | 9.85 | 9.27 | -3.63 | 7.63 |
| Education: tertiary level |  |  | 22.28 | 11.45 | 16.33 | 9.10 | -7.81 | 7.48 |
| Education: Bachelor |  |  | 28.73* | 11.67 | 14.54 | 9.23 | -13.05 | 7.65 |
| Education: Master |  |  | 29.86* | 12.03 | 17.45 | 9.19 | -11.98 | 7.89 |
| Education: PhD |  |  | 32.31* | 12.70 | 15.82 | 9.37 | -14.22 | 8.34 |
| Significance level: **P* ≤ .05; ** *P* < .01; *** *P* < .001; β: estimated beta-values; se: standard errors  ^a^Mean score range from 0 (do not agree at all) to 100 (fully agree) | | | | | | | | |
