# Supplementary material for: Technostress and Digital Competence Among Health Professionals in Swiss Psychiatric Hospitals: Cross-sectional Study
Source: JMIR Ment Health. 2021 Nov 4;8(11):e31408. doi: 10.2196/31408 (PMC8603177; doi:10.2196/31408)
Supplement: Multimedia Appendix 3 [file mental_v8i11e31408_app3.docx]

Table 4: Multiple linear regression models with long-term consequences as outcomes part 2 (observations n=493)

|  | General health status^b^ | | Quality of sleep^a^ | | Headache^c^ | | Work-ability^d^ | |
| --- | --- | --- | --- | --- | --- | --- | --- | --- |
|  |  | |  | |  | |  | |
|  | R2 = 0.06, F(3,489) = 9.88, *P* < .001 | | R2 = 0.08, F(3,489) = 13.25, *P* < .001 | | R2 = 0.10, F(5,487) = 11.07, *P* < .001 | | R2 = 0.12, F(9,483) = 7.64, *P* < .001 | |
|  | β | se | Β | se | β | se | β | se |
| Intercept | 95.26*** | 3.68 | 61.37*** | 5.48 | 23.43** | 7.43 | 37.84*** | 1.49 |
| Technostress | -4.47*** | 1.33 | -5.87*** | 1.60 | 6.58*** | 1.88 | -1.40*** | 0.34 |
| Digital Competence |  |  | 4.19*** | 1.15 | -3.00* | 1.36 | 0.79** | 0.26 |
| Sex: male |  |  |  |  | -8.38*** | 2.18 | 0.79 | 0.41 |
| Work experience | 0.12 | 0.06 |  |  |  |  |  |  |
| Age |  |  | 0.17* | 0.07 | -0.32*** | 0.08 | 0.04** | 0.015 |
| Level of employment | -0.15*** | 0.39 |  |  | 0.13* | 0.06 | -0.03* | 0.01 |
| Physicians |  |  |  |  |  |  | 0.80 | 0.81 |
| Psychologists |  |  |  |  |  |  | 0.77 | 0.73 |
| Nurses |  |  |  |  |  |  | -0.70 | 0.59 |
| Medical therapeutic professionals |  |  |  |  |  |  | -0.30 | 0.83 |
| Significance level: * *P* ≤ .05; ** *P* < .01; *** *P* < .001; β: estimated beta-values; se: standard errors  ^a^Mean score ranges from 0 (“do not agree at all”) to 100 (“fully agree”),  ^b^Mean score ranges from 0 (“worst imaginable health state”) to 100 (“best imaginable health state”),  ^c^Mean score ranges from 0 (no influence) to 100 (could no longer perform activity),  ^d^Total score ranges from 7 (minimum working capacity) to 49 (maximum working capacity). | | | | | | | | |
